# Camphene, a Plant Derived Monoterpene, Exerts Its Hypolipidemic Action by Affecting SREBP-1 and MTP Expression

**DOI:** 10.1371/journal.pone.0147117

**Published:** 2016-01-19

**Authors:** Ioanna Vallianou, Margarita Hadzopoulou-Cladaras

**Affiliations:** Department of Genetics, Development and Molecular Biology, School of Biology, Aristotle University of Thessaloniki, Thessaloniki, 54124, Greece; University of Basque Country, SPAIN

## Abstract

The control of hyperlipidemia plays a central role in cardiovascular disease. Previously, we have shown that camphene, a constituent of mastic gum oil, lowers cholesterol and triglycerides (TG) in the plasma of hyperlipidemic rats without affecting HMG-CoA reductase activity, suggesting that its hypocholesterolemic and hypotriglyceridemic effects are associated with a mechanism of action different than that of statins. In the present study, we examine the mechanism by which camphene exerts its hypolipidemic action. We evaluated the effect of camphene on the *de novo* synthesis of cholesterol and TG from [^14^C]-acetate in HepG2 cells, along with the statin mevinolin. Camphene inhibited the biosynthesis of cholesterol in a concentration-dependent manner, and a maximal inhibition of 39% was observed at 100 μM while mevinolin nearly abolished cholesterol biosynthesis. Moreover, treatment with camphene reduced TG by 34% and increased apolipoprotein AI expression. In contrast, mevinolin increased TG by 26% and had a modest effect on apolipoprotein AI expression. To evaluate the mode of action of camphene, we examined its effects on the expression of SREBP-1, which affects TG biosynthesis and SREBP-2, which mostly affects sterol synthesis. Interestingly, camphene increased the nuclear translocation of the mature form of SREBP-1 while mevinolin was found to increase the amount of the mature form of SREBP-2. The effect of camphene is most likely regulated through SREBP-1 by affecting MTP levels in response to a decrease in the intracellular cholesterol. We propose that camphene upregulates SREBP-1 expression and MTP inhibition is likely to be a probable mechanism whereby camphene exerts its hypolipidemic effect.

## Introduction

High levels of plasma cholesterol and triglycerides are strongly associated with the development of atherosclerosis and coronary heart disease (CHD) [1–4]. Clinical trials designed to reduce serum cholesterol levels by diet or pharmacological intervention have resulted in marked reduction of disease incidence [5]. However, the search for drugs to treat dislipidemia, still remains a major pharmaceutical focus.

Various lipid-modifying drugs with different mechanisms of action have been developed and used for the treatment of patients with dislipidemia. As over 70% of cholesterol in the body is derived from *de novo* cholesterol biosynthesis, inhibition of this biosynthesis presents an effective therapeutic approach to reduce plasma cholesterol. Clinically, statins have been the most widely prescribed drugs for the treatment of hypercholesterolemia [6, 7]. In particular, statins are competitive inhibitors of 3-hydroxy-3-methylglutaryl coenzyme A (HMG-CoA) reductase, the rate limiting enzyme in cholesterol biosynthesis [8]. Inhibition of cholesterol biosynthesis leads to decreased intracellular cholesterol concentrations and activation of the Sterol Regulating Binding Protein-2 (SREBP-2), thereby resulting in up-regulation of the Low Density Lipoprotein (LDL) receptor and a subsequent lowering of LDL cholesterol in blood as a consequence of increased LDL clearance [9]. However, despite the therapeutic success of statins, there is a need of new therapeutical interventions to reduce cholesterol, since some patients do not tolerate statins well and more importantly many patients under statin treatment alone do not achieve the LDL cholesterol goal according to guidelines suggested by the National Institute of Health, USA. In addition to testing new approaches to LDL cholesterol lowering there has been a major focus on treatments that can favorably influence High Density Lipoprotein (HDL) and triglycerides concentrations. Also, alternative approaches to treat dyslipidemia are being developed and are based on the inhibition of enzymes involved in lipid metabolism such as Acyl-CoA:cholesterol acyltransferase (ACAT), microsomal triglyceride transfer protein (MTP), 2,3-oxidosqualene:lanosterol cyclase (OSC), cholesteryl ester transport protein (CETP) and others [10, 11]. Recently, several efforts aim to develop more effective hypolipidemic agents that originate from natural agents. We have previously shown that camphene, a plant monoterpene derived from chios mastic gum oil (MGO), decreases serum total cholesterol, LDL cholesterol and triglyceride levels in hyperlipidemic rats [12]. Camphene represents only a minor constituent of MGO (0.83%) and the hypolipidemic activity of MGO was associated with camphene [12]. We have also reported that camphene treatment led to a decrease in cellular cholesterol and cholesterol ester content to the same extend as statins and exhibited no toxicity in human hepatic cells. Moreover, the lipid-lowering action of camphene was found to be independent of HMG-CoA reductase activity, suggesting that the hypolipidemic effects of camphene are mediated through a mechanism of action different to that of statins [12]. Camphene is a bicyclic monoterpene (2,2-dimethyl-3-methylene-bicyclo[2,2,1] heptane, C_10_H_16_) and a constituent of many essential oils derived from various plants [13–19]. Camphene was also reported to prevent hepatic steatosis and improve lipid accumulation in high fat diet mice [20]. Recent studies have shown that camphene presents a protective effect against oxidative stress [16] and incubation of human plasma with camphene protects LDL against copper induced oxidation [21]. Hence, the purpose of this study was to further investigate the molecular mechanism of the hypolipidemic activity of camphene.

Apart from elevated plasma LDL, low levels of HDL are a known risk factor for the development and progression of atherosclerosis and CHD [22, 23]. HDL cholesterol has a well documented anti-atherogenic involvement. The cardioprotective effect of HDL has been attributed mostly, but not exclusively, to its major protein constituent, namely apolipoprotein AI (apoAI) [24]. Apolipoprotein AI is the critical protein component of HDL particles and is responsible for reverse cholesterol transport [25], transporting cholesterol from peripheral tissues to the liver for further metabolism and excretion. Some epidemiological studies confirm the inverse correlation between serum apoAI concentrations and cardiovascular events [26–29]. Considering the high prevalence of low HDL in patients with CHD, therapeutic increases in HDL are an obvious approach to decreasing the risk of atherosclerosis.

There is accumulating evidence that plasma triglyceride is an independent risk factor in CHD [30, 31]. High levels of triglycerides are very often associated with low levels of HDL. Statins have little effect on plasma triglyceride levels [32, 33] and agents that could reduce both cholesterol and plasma triglyceride levels would therefore be highly desirable.

Sterol regulatory element-binding proteins (SREBPs) are major transcription factors that control the expression of crucial genes involved in lipogenesis and lipid uptake. SREBP-1a and -1c are primarily responsible for the genes involved in the biosynthesis of cholesterol and fatty acids, whereas SREBP-2 mainly mediates the biosynthesis of cholesterol [34, 35]. Targeting SREBPs can be a novel strategy to treat atherosclerosis.

In the present study, we have investigated the mode of action of camphene. We found that camphene treatment not only reduced fatty acid, triglyceride as well as cholesterol *de novo* biosynthesis in HepG2 cells but also had an effect on apoAI protein levels, the major component of HDL, and SREBP transcription factors, the master regulators of genes that are responsible for lipid biosynthesis. The hypolipidemic effect of camphene is probably exerted through MTP inhibition.

## Materials and Methods

### Materials

Camphene, mevinolin, atorvastatin and U18666A were from Sigma-Aldrich. [2-^14^C] acetic acid sodium salt (250 μCi) and [1-^14^C] oleic acid (50 μCi) were purchased from Amersham Biosciences. ACAT inhibitor, F1394, was kindly provided by Dr Constantinos G. Panousis. Reagents for protein determination were from Bio-Rad. The human hepatic cell line, HepG2, was purchased from ATCC. Cell culture medium, Dulbecco’s modified Eagle’s medium (DMEM), fetal bovine serum (FBS), and antibiotics, penicillin and streptomycin, were from Gibco BRL Life Technologies. Lipoprotein-deficient serum (LPDS) was obtained from Autogen Bioclear UK Ltd. TLC plates were from Macherey Nagel.

### Cell culture

HepG2 cells were cultured in DMEM supplemented with 10% FBS, penicillin (100 units/ml) and streptomycin (0.25 μg/ml) at 37°C in a humidified atmosphere of 5% CO_2_. The cell cultures were routinely passaged, while growing exponentially. The test compounds were dissolved in absolute ethanol or DMSO prior to appropriate dilution with cell culture medium so that the final ethanol or DMSO concentration never exceeded 0.5% (v/v) in the culture.

### Cell treatment with various compounds and preparation of cell extracts

On day 0, 1.5x10^5^ cells were seeded in cell culture dishes (60-mm diameter) in DMEM containing 10% FBS. On day 3 or 4, the medium was replaced with FBS-containing fresh medium, which was subsequently replaced on day 6 with DMEM containing 10% LPDS, instead of FBS, followed by incubation for 24 h. On day 7, the cells were incubated for 24 or 48 h with camphene and mevinolin in fresh DMEM containing 10% LPDS. Following incubation, the cells were washed three times with cold PBS, pH 7.4, and detached from the substrate with the aid of a rubber policeman. PBS containing the detached cells was centrifuged at 3000 rpm for 5 min at 4°C, the clarified supernatant was discarded, and the cell pellet was stored at -80°C until used.

Cell extracts were prepared according to a published procedure [36]. Briefly, the cell pellet was suspended in 100 μl of a lysis buffer containing 50 mM imidazole, pH 7.4, 250 mM NaCl, 2 mM EGTA, 1 mM EDTA, 5 mM dithiothreitol, 50 μM leupeptine and 0.25% Brij 97. After incubation for 30 min at 37°C the homogenate was centrifuged for 10 min at 12000 rpm at 4°C and aliquots of the supernatant were used to assay cellular protein content and for Western blot analysis. The cellular protein content was determined by Bradford’s method using the Bio-Rad protein assay.

### Immunoblotting analysis

After incubation, cells were washed with PBS and then treated with lysis buffer as described above. Cell protein content was determined, loaded equally (25 μg protein/lane) and separated on SDS polyacrylamide gels (4% stacking, 12% separating for apoAI and 8% separating for SREBP-1 and SREBP-2). Proteins were then electroblotted onto polyvinylidene fluoride membranes (Amersham) and nonspecific binding sites were blocked for 1 h at room temperature by 5% (w/v) non fat milk (Blotto, non-fat dry milk, Santa Cruz) in PBS containing 0.1% Tween-20 and 0.5% BSA (bovine serum albumin, fraction V, Applichem). The blots were incubated with specific primary antibodies against apoAI (1:5000; 23698, Polysciences Inc.), SREBP-1 (1:1000; sc-8984, Santa-Cruz), SREBP-2 (1:1000; sc-8151, Santa-Cruz) or MTP (1:200, sc-33116, Santa-Cruz) for 1 h at room temperature. Anti-mouse actin antibody (1:2000, sc-47778, Santa-Cruz) was used as a loading control. Primary antibodies were detected with horseradish peroxidase-conjugated secondary antibodies (Santa Cruz). The specific protein bands were visualized with the enhanced chemiluminescence (ECL) plus Western blotting detection system kit (Amersham Biosciences) according to the instructions of the manufacturer. The intensities of the various protein bands were quantified using Phosphorimager. To inhibit rapid degradation of nuclear fragments of SREBPs cells were treated with N-acetyl-leucinal-norleucinal (ALNN) (25 μg/ml) for the last 5 hours before protein extraction and throughout the extraction procedure [37].

### Preparation of [^14^C]-acetate and [^14^C]-oleate/albumin complex solution

Sodium [^14^C]-acetate in ethanol was evaporated to dryness and resuspended in a solution of non radioactive sodium acetate (1 mM) to the indicated specific activity (57mCi/mmol). [^14^C]-oleate in hexane was evaporated to dryness and resuspended in a solution containing non-radioactive sodium oleate (4 mM) in complex with 6% (w/v) BSA (fraction V, Applichem). Oleic acid-BSA complex was prepared as follows. Twenty μmoles sodium oleate was dissolved in chloroform:methanol, 1:2 v/v and the solvent was then evaporated to dryness. Five μmoles BSA was added in DMEM prewarmed at 37°C. The oleic acid solution was then added to the BSA solution drop wise while stirring and the solution was incubated at 37°C for 1–2 hours until dissolved. The oleic acid/albumin solution prepared was optically clear. The molar ratio of oleic acid to albumin was kept at 4:1.

### Analysis of cholesterol biosynthesis and lipid determinations

Lipid synthesis was determined by measuring the incorporation of [^14^C]-acetate into lipids according to the method of Brown *et al* with some modifications [38]. On day 7, the cells were incubated for 24 h in the presence of vehicle or compounds (camphene, mevinolin, atorvastatin, F1394 or U18666A) at the indicated concentrations in fresh DMEM containing 10% LPDS. Four hours before the end of the incubation period, cells were washed three times with serum-free DMEM and incubated at 37°C in the serum free DMEM medium supplemented with 1μCi/ml [^14^C]-acetate (1 mM) (for fatty acid, TG and cholesterol synthesis) or 0.5 μCi/ml [^14^C]-oleic acid (0.4 mM) (for assessing fatty acid esterification to produce TG) and the above mentioned compounds at the indicated concentrations. After pulse-labeling for 4 h, the medium was removed and cells were washed thoroughly with PBS. Total cellular lipids were extracted by the method of Bligh and Dyer [39]. Briefly, appropriate volumes of chloroform and methanol were added to cells suspended in 1 ml of PBS in order to obtain a one-phase system consisting of CHCl_3_/CH_3_OH/H_2_O (1:2:0.8, v/v/v). After vigorous mixing, the appropriate volumes of chloroform and water were added to obtain a two-phase system CHCl_3_/CH_3_OH/H_2_O at 1:1:0.9 ratios (v/v/v) followed by another vigorous mixing. Then, the mixture was allowed to equilibrate for 30 min at –20°C until the two phases were separated. The chloroform phase containing total lipids was removed and evaporated under nitrogen stream and lipid extract was reconstituted in 100 μl hexane. Radiolabeled lipids were spotted on silica coated plates and separated by thin-layer chromatography (TLC) using hexane/diethyl ether/acetic acid (80:20:1, v/v/v) as a developing solvent. Lipids were identified by using purified standards (eg fatty acids, cholesterol and TG) and the radioactivity associated with each individual lipid was quantified using Phosphorimager.

### RNA isolation, cDNA synthesis and RT-qPCR (quantitative real-time reverse transcription PCR)

Total RNA was extracted from HepG2 cells using the RNeasy kit (Qiagen) and underwent quantitative analysis of mRNA expression of MTP by use of real time PCR (StepOne, Appied Biosystems). cDNA was synthesized from 1 μg RNA using QuantiTect Reverse Transcription Kit (Qiagen). The reaction reagent SYBR Green Master mix kit (Kapa SYBR Green qPCR, Kapa Biosystems) was used according to the manufacturer’s instructions. Gene-specific mRNA was normalized to YWHAZ and actin as internal controls. The amounts of mRNAs in camphene treated cells were expressed as fold change relative to those of control-untreated cells set as 1. All real time PCRs were run in duplicate. Sequences of the primers sets used were as follows:

**MTP:** 5’- ACA AGC TCA CGT ACT CCA CTG-3’ (forward) and 5’- TCC TCC ATA GTA AGG CCA CAT C-3’ (reverse)

**YWHAZ:** 5’- GCT GGT GAT GAC AAG AAA GG-3’ (forward) and 5’-GGA TGT GTT GGT TGC ATT TCC T-3’ (reverse)

**actin:** 5’-CCA ACC GCG AGA AGA TGA-3’ (forward) and 5’- CCA GAG CGG TAC AGG GAT AG-3’ (reverse).

### Statistical analysis

Mean and standard error of means are reported. Significant differences between values of treated and control groups were determined using the Student-Newmann-Keuls test.

## Results

### Effect of camphene on HepG2 lipid profile

**Fig 1:**
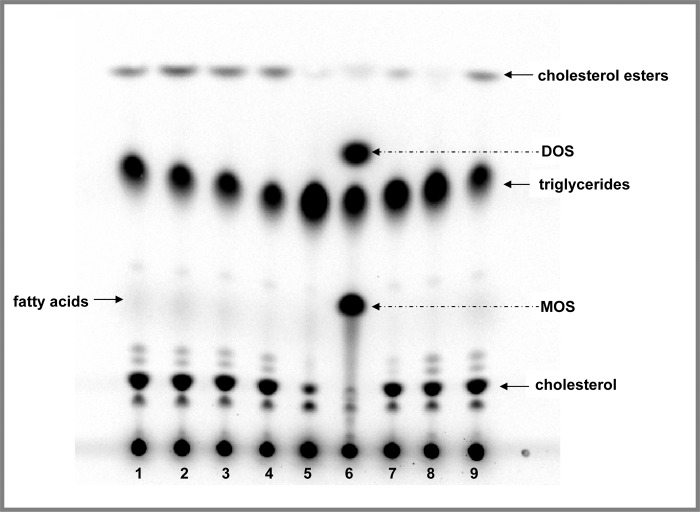
Effect of camphene on lipid profile and metabolism in HepG2 cells-Comparison with well known hypolipidemic compounds. On day 7, the cells were incubated with the compounds for 24 h in DMEM containing 10% (v/v) human LPDS. HepG2 cells were treated with camphene (lanes: 2–4), mevinolin (lane: 5), atorvastatin (lane: 7), the OSC inhibitor, U18666A (lane: 6) and the ACAT inhibitor, F1394 (lane: 8). Control-non treated cells migrate in places 1 and 9. Camphene was used at concentration of 25, 50 and 100 μM lanes 2, 3 and 4 respectively. Four hours prior to the end of the incubation period, the cells were pulse-labeled with [^14^C]-acetic acid, sodium salt. This panel shows the autoradiograph of synthesized intracellular lipids separated by TLC after labelling of cells with [^14^C]-acetate. The positions of the different lipids have been determined using non radioactive standards. The arrows indicate the positions of migration of cholesterol, fatty acids, triglycerides, cholesterol esters, MOS and DOS.

We used HepG2 cells to explore the potential of camphene to modify lipid metabolism. HepG2 cells were chosen for the present study since they have been found to possess a wide variety of liver specific metabolic functions including lipoprotein synthesis and lipid metabolism and are particularly suitable to study cholesterol and triglyceride biosynthesis [40]. Cells were pulse-chased with [^14^C]-acetate to label the full panel of lipids and the effect of camphene on HepG2 lipid profile was compared with that of other lipid lowering agents that are known to affect lipid metabolism. The well known HMG-CoA reductase inhibitors, mevinolin and atorvastatin, were used as positive controls to inhibit cholesterol and cholesterol ester biosynthesis in hepatocytes. The effect of camphene on lipid profile was also compared to that of the ACAT inhibitor, F1394, and the OSC inhibitor, U18666A. A representative autoradiograph of lipid profiles under different compound treatments is shown in Fig 1. As it was expected mevinolin and atorvastatin caused a dramatic decrease in cholesterol and cholesterol ester levels. The ACAT inhibitor, F1394, inhibited the conversion of cholesterol to cholesterol esters and led to a decrease in cholesterol ester levels. The OSC inhibitor, U18666A, in addition to the well known bands corresponding to cholesterol, cholesterol ester and triglycerides, induced two additional bands which correspond to 2,3-monoepoxysqualene (MOS) and 2,3;22,23-diepoxysqualene (DOS). OSC is a rate limiting enzyme that catalyzes the conversion of MOS to lanosterol. MOS accumulation that results from OSC inhibition is visualized after treatment with OSC inhibitor. When the conversion of MOS is inhibited part of the MOS that accumulates can be converted into DOS. As shown in Fig 1, camphene did not induce a similar profile neither to OSC inhibitor as the two additional bands of MOS and DOS were not observed, nor to ACAT inhibitor as it did not seem to affect cholesterol ester levels. However, camphene treatment appeared to decrease triglyceride levels.

### Effect of camphene on the *de novo* biosynthesis of cholesterol, fatty acids and triglycerides

**Fig 2:**
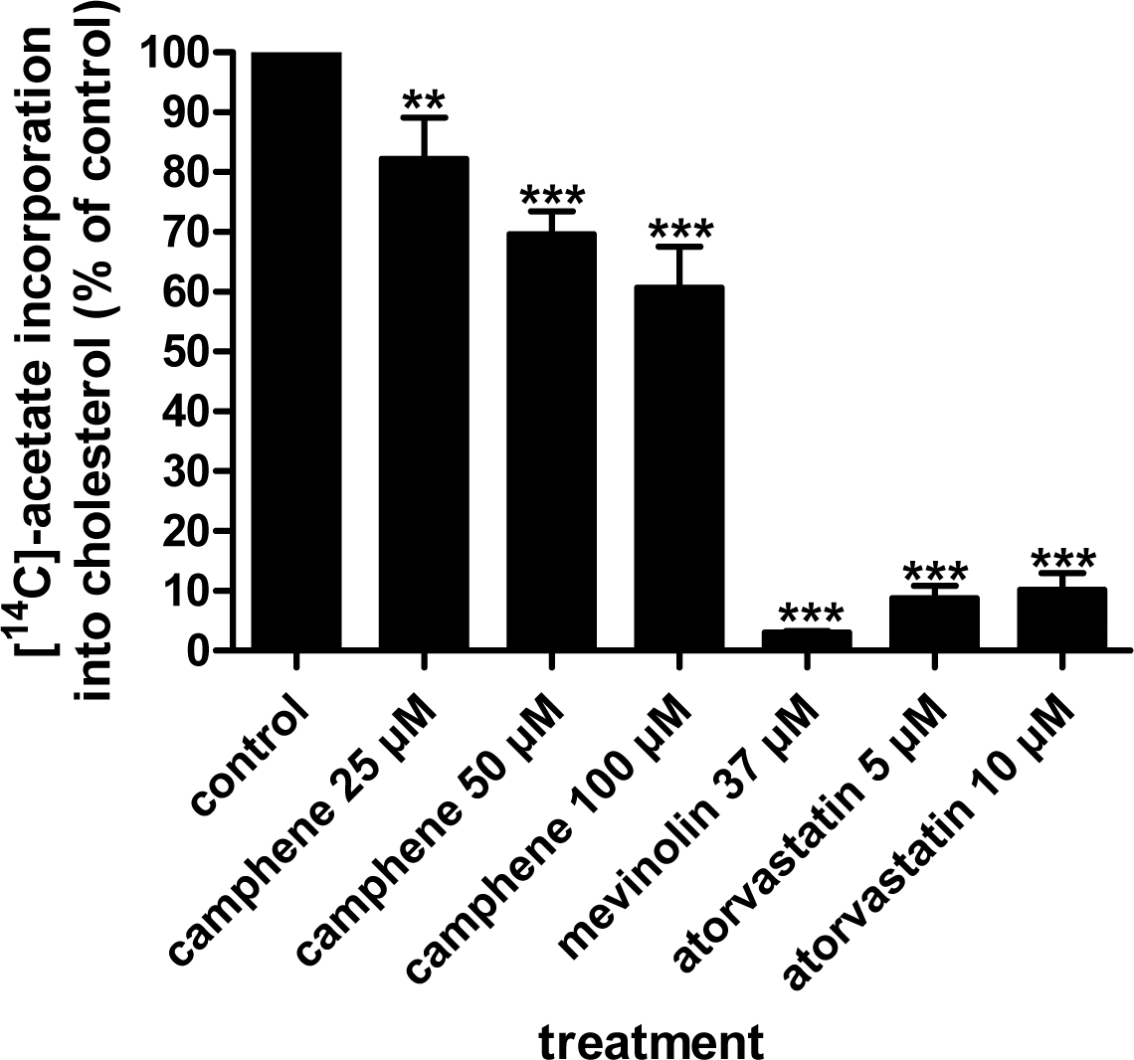
Effect of camphene, mevinolin and atorvastatin on the incorporation of acetate into *de novo* synthesized cholesterol. On day 7, the cells were incubated with the compounds for 24 h in DMEM containing 10% (v/v) human LPDS. Camphene, mevinolin and atorvastatin were received at the concentrations indicated. Four hours prior to the end of the incubation period, the cells were pulse-labeled with [^14^C]-acetic acid, sodium salt. The incorporation of acetate into cholesterol was determined as described. Results were normalized to the amount of cellular protein and expressed in percent of the respective control incubations. Results are from three independent experiments in triplicates and expressed as the mean ± SD. Significantly different compared to control: *p<*0.05 (*); *p<*0.01(**); *p<*0.001 (***) vs control by Student-Newmann-Keuls test. 25 μM vs 50 μM *p*<0.05; 25 μM vs 100 μM *p*<0.01; 50 μM vs 100 μM *p*>0.05.

To analyze and quantify the levels of neo-synthesized lipids, HepG2 cells were incubated with camphene, mevinolin and atorvastatin and the incorporation of [^14^C]-acetate into cholesterol, fatty acids and triglycerides was determined. In order to upregulate the mevalonate pathway, cells were preincubated with medium containing LPDS. Camphene inhibited the incorporation of [^14^C]-acetate into newly synthesized cholesterol in a concentration dependent manner (Fig 2). Camphene at 25 μM inhibited cholesterol biosynthesis by 18% (*p*<0.01) compared with the control, while at 50 μM decreased *de novo* synthesis of cholesterol by 30% (*p*<0.001). The maximum inhibition of cholesterol production, 39%, was induced by 100 μM camphene (*p*<0.001). As it was expected, the statins, mevinolin and atorvastatin, nearly abolished [^14^C]-acetate conversion into cholesterol biosynthesis from the precursor [^14^C]-acetate as they decreased newly synthesized cholesterol levels by 97% and 90% respectively (*p*<0.001). Therefore, camphene significantly inhibits cholesterol biosynthesis, but not to the same potent extent as statins do.

**Fig 3:**
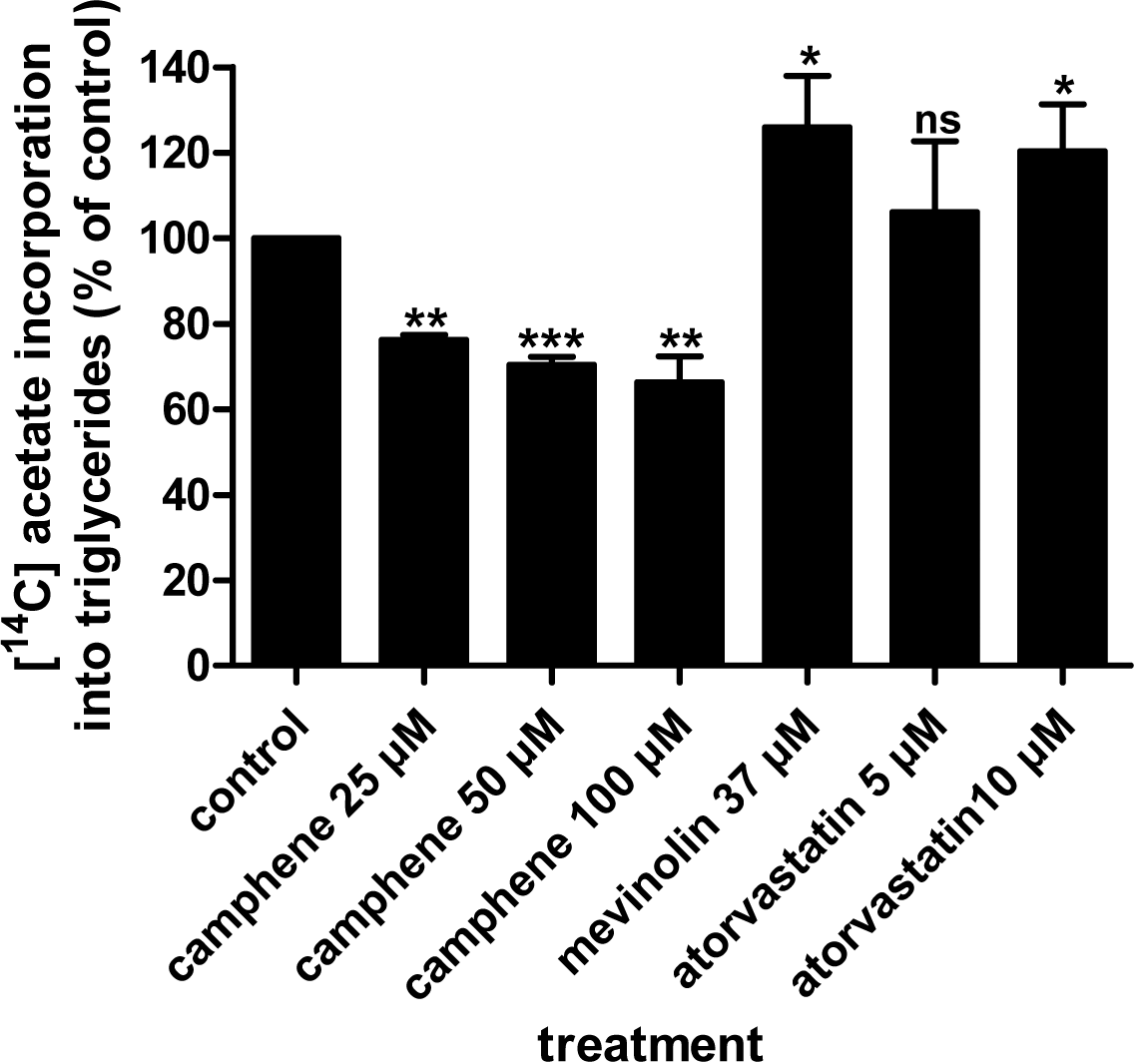
Effect of camphene, mevinolin and atorvastatin on the incorporation of [^14^C]-acetate into *de novo* synthesized triglycerides. On day 7, HepG2 cells were incubated for 24 h with each compound in medium containing 10% (v/v) human LPDS. The cells received camphene, mevinolin and atorvastatin at the concentrations indicated. Four hours prior to the end of the incubation period, the cells were pulse-labeled with [^14^C]-acetic acid, sodium salt. The incorporation of acetate into triglycerides was determined as described in Materials and Methods section. Results were normalized to the amount of cellular protein and expressed in percent of the respective control incubations. Results are from three independent experiments in triplicates and expressed as the mean ± SD. Significantly different compared to control: *p*<0.05 (*); *p*<0.01 (**); *p*<0.001 (***); *p>*0.05 and ns (non significant) vs control by Student-Newmann-Keuls test.

Incubation of cells with varying concentrations of camphene inhibited triglyceride synthesis as determined by the incorporation of [^14^C]-acetate into triglycerides. Treatment of HepG2 cells with 25 μM, 50 μM and 100 μM camphene inhibited triglyceride biosynthesis by 24% (*p*<0.01), 30% (*p*<0.001) and 34% (*p*<0.01) respectively, compared to untreated cells (Fig 3). There were no significant differences between the effects of different concentrations of camphene. Interestingly, mevinolin and atorvastatin increased the amount of newly synthesized triglycerides by 26% (*p*<0.05) and 20% (*p*<0.05) respectively. Again, there were no significant changes between the effects of mevinolin and atorvastatin. However, camphene effect on the *de novo* triglyceride biosynthesis was significantly different from that of statins. Camphene inhibited the incorporation of [^14^C]-acetate into triglycerides suggesting decreased *de novo* synthesis of triglycerides.

**Fig 4:**
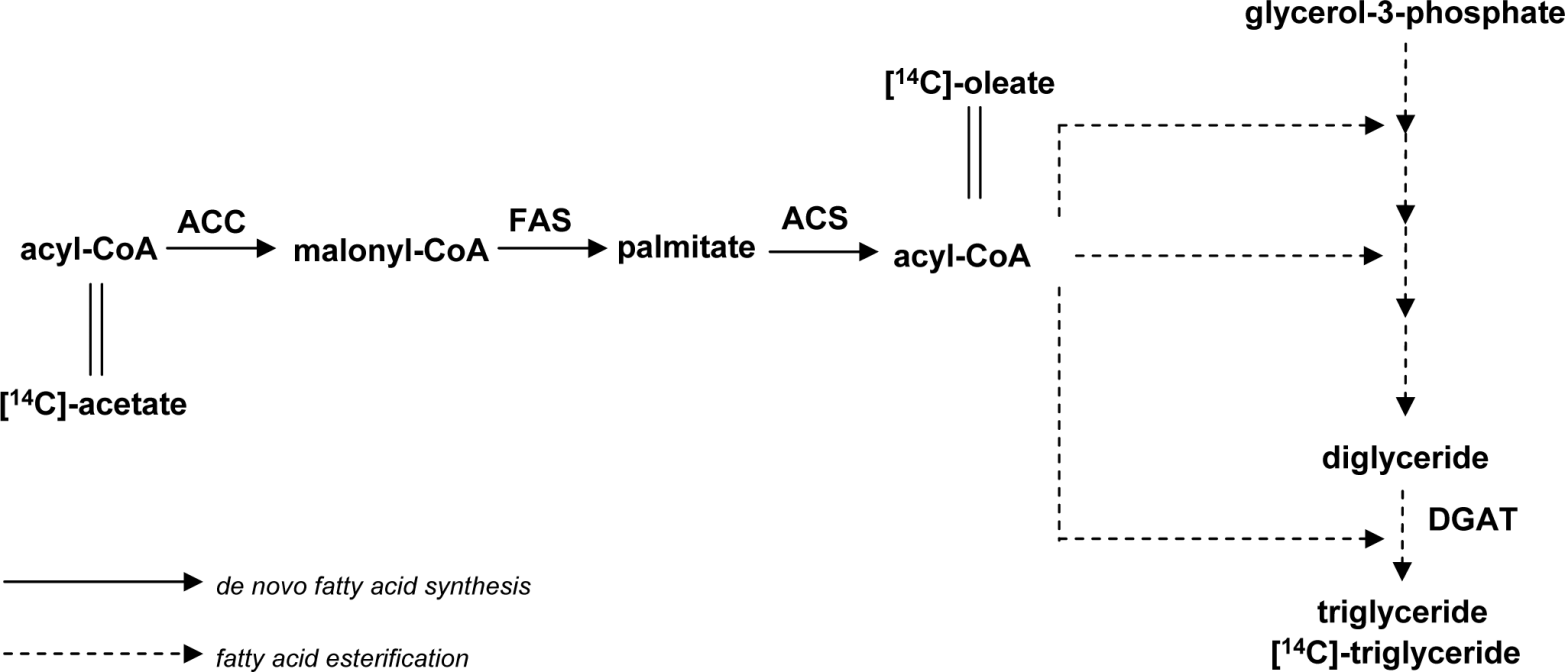
Incorporation of [^14^C]-acetate and [^14^C]-oleate in the biosynthetic pathway of triglycerides.

**Fig 5:**
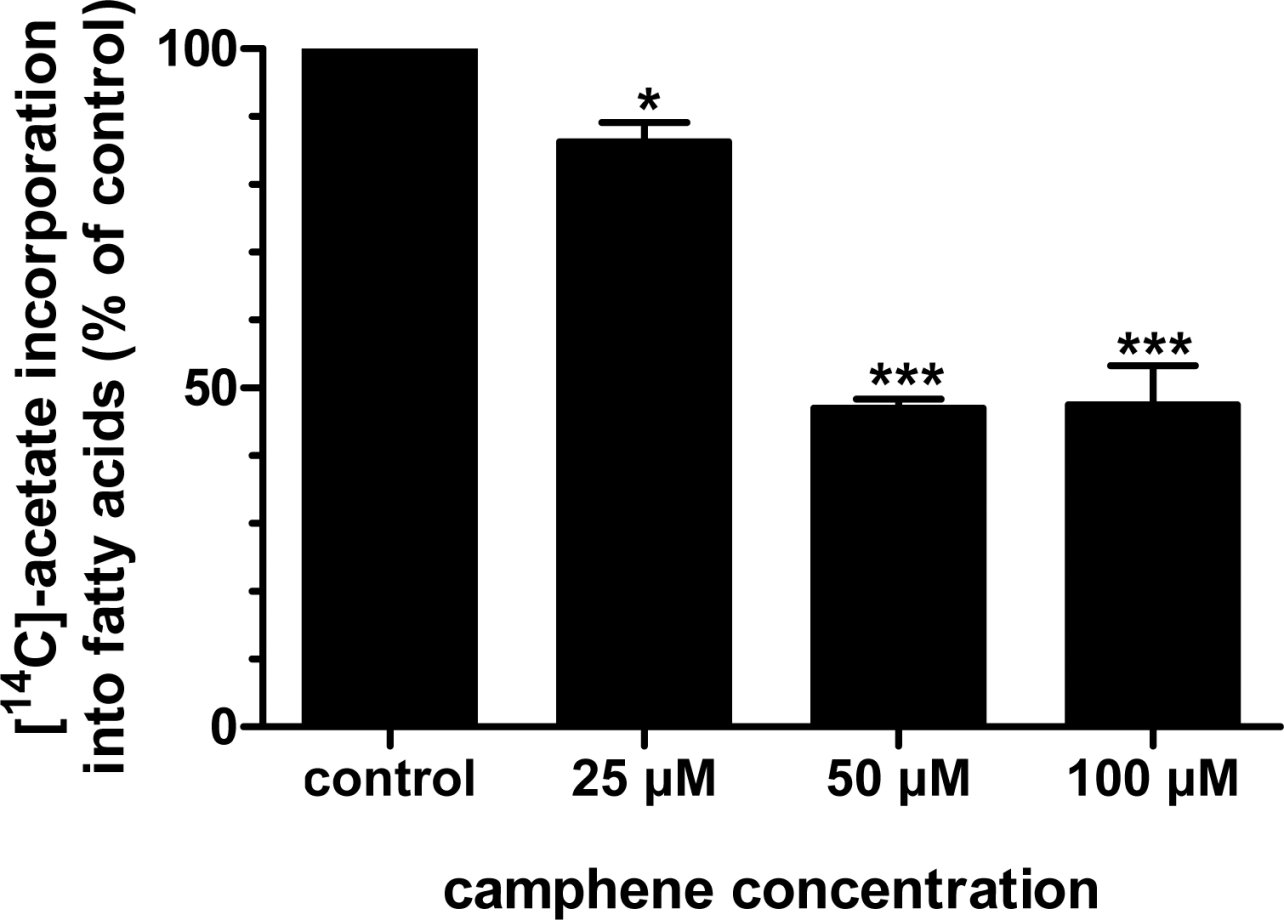
Effect of camphene on the incorporation of [^14^C]-acetate into *de novo* synthesized fatty acids. On day 7, HepG2 cells were incubated for 24 h with camphene (25, 50 and 100 μM) in medium containing 10% (v/v) human LPDS. Four hours prior to the end of the incubation period, the cells were pulse-labeled with [^14^C]-acetic acid, sodium salt. The incorporation of acetate into fatty acids was determined as described. Results were normalized to the amount of cellular protein and expressed in percent of the respective control incubations. Results are from three independent experiments in triplicates and expressed as the mean ± SD. Significantly different compared to control: *p*<0.05 (*); *p* <0.001 (***) and ns (non significant) vs control by Student-Newmann-Keuls test.

To elucidate the mechanism of inhibition of triglyceride synthesis by camphene, we examined total fatty acid synthesis from [^14^C]-acetate and triglyceride synthesis from [^14^C]-oleate (Fig 4). Camphene significantly reduced *de novo* fatty acid biosynthesis as monitored by the incorporation of [^14^C]-acetate into fatty acids. In particular, camphene at the lowest concentration analyzed, 25 μM, reduced fatty acid biosynthesis by 14% (*p*<0.05). Significant reductions in fatty acid biosynthesis were observed at concentrations 50 and 100 μM, as the production of fatty acids was reduced by 53% (*p*<0.001) and 52.5% (*p*<0.01) respectively (Fig 5).

**Fig 6:**
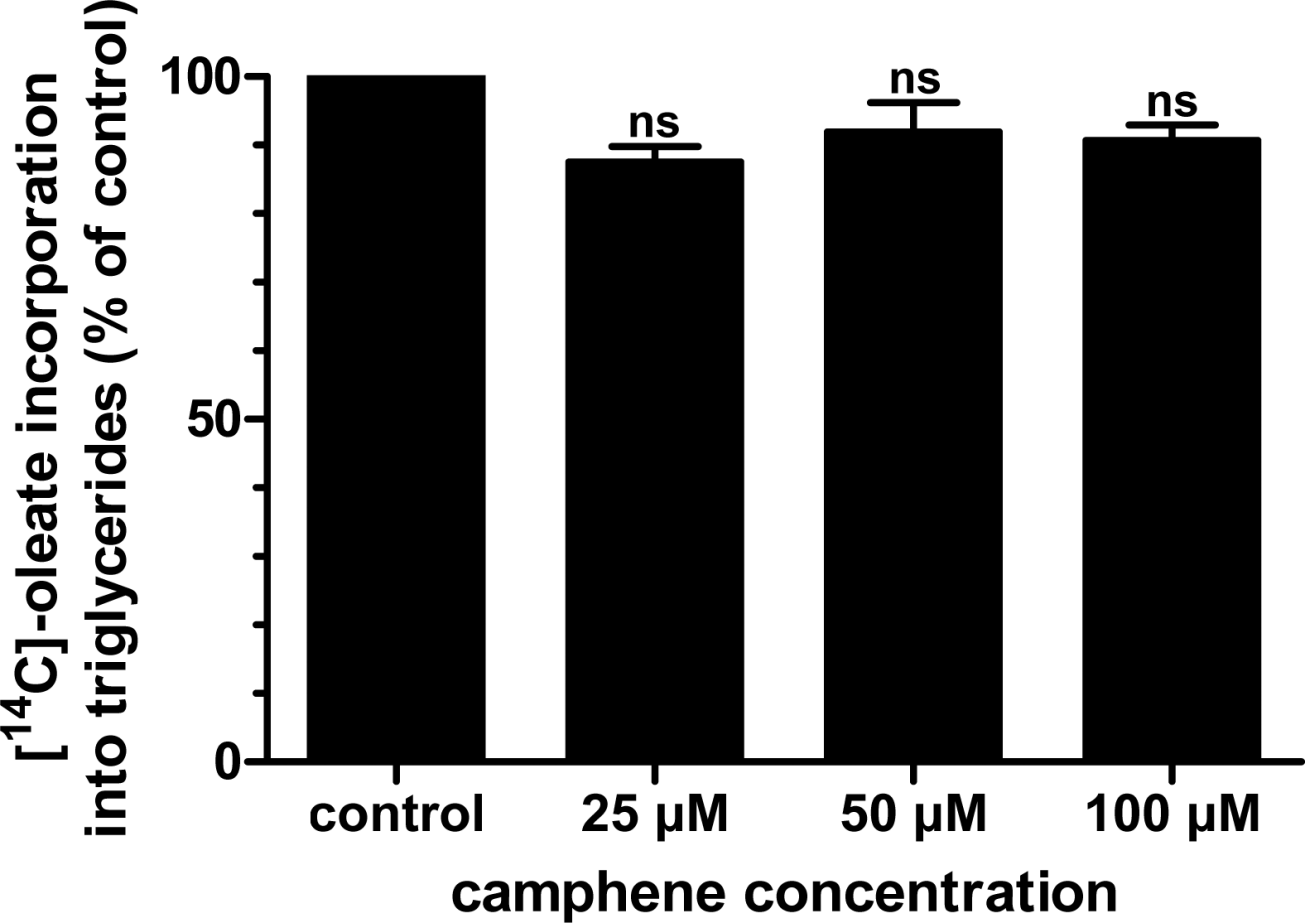
Effect of camphene on the esterification of fatty acids into triglycerides. On day 7, HepG2 cells were incubated for 24 h with each compound in medium containing 10% (v/v) human LPDS. The cells received camphene (25, 50 and 100 μM), mevinolin (37 μM) or atorvastatin (10 μM). Four hours prior to the end of the incubation period, the cells were pulse-labeled with [^14^C]-oleic acid, sodium salt. The incorporation of oleate into triglycerides was determined as described. Results were normalized to the amount of cellular protein and expressed in percent of the respective control incubations. Results are from three independent experiments in triplicates and expressed as the mean ± SD. Results not statistically significant vs control, ns (non significant) by Student-Newmann-Keuls test.

As camphene inhibited the synthesis of both fatty acids and triglycerides, we further examined whether camphene has a significant role in the esterification of fatty acids to form triglycerides. These studies were performed by examining the incorporation of radiolabeled oleic acid into triglycerides (Fig 4). The results indicated that incubation of HepG2 cells with camphene did not significantly alter oleic acid esterification to form triglycerides (Fig 6). Mevinolin and atorvastatin had also no effect on the incorporation of [^14^C]-oleate into the triglyceride fraction (results not shown). Therefore, camphene inhibited total fatty acid synthesis from acetate (*de novo* fatty acid synthesis). However, camphene had no effect on the incorporation of [^14^C]-oleate into the triglyceride fraction up to 100 μM.

### Comparative effects of camphene and mevinolin on apoAI protein levels

**Fig 7:**
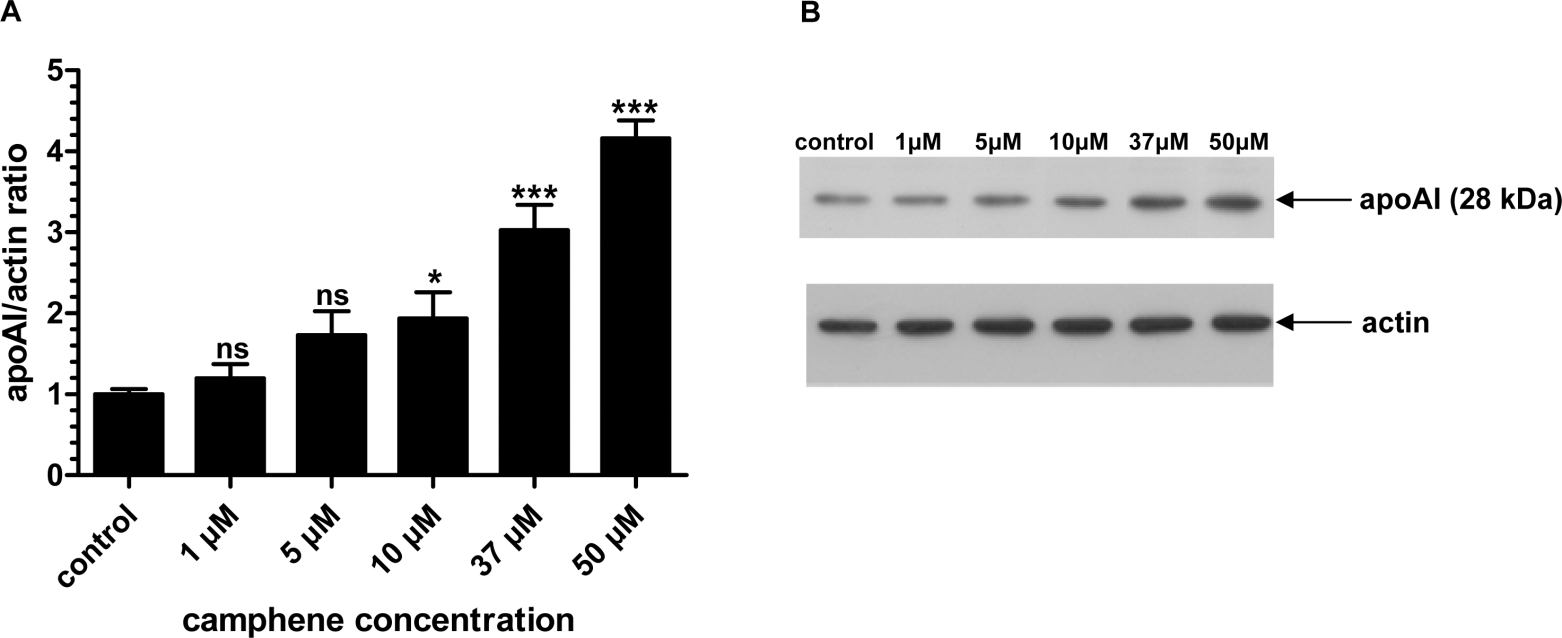
Effect of camphene on the protein expression of apoAI. **Panel A.** On day 7, HepG2 cells were incubated for 48 h with camphene (1, 5, 10, 37 and 50 μM) in DMEM containing 10% LPDS. Total cell protein was extracted and 50 μg of protein were subjected to SDS PAGE electrophoresis and analyzed by Western immunoblotting using goat anti-apoAI antibody as described. Mouse β-actin was used to control for equal loading and normalization. Relative intensity of the bands was quantified using Phosphorimager. Values express the apoAI/actin ratio and are calculated by comparison to control samples the value of which is defined as 1. Values are means ± SD of three independent experiments in triplicates. *p<*0.05 (*); *p<*0.001 (***); *p>*0.05 and ns (non significant) vs control by the Student-Newmann-Keuls test. 1 μM is significantly different from 37 and 50 μM with *p*<0.01 and *p*<0.001 respectively. 5 μM treatment is significantly different from 37 and 50 μM with *p*<0.05 and *p*<0.001 respectively. 10 μM treatment is significantly different from 37 and 50 μM treatments with *p*<0.05 and *p*<0.001 respectively. Camphene concentration of 37 μM is significantly different from 50 μM with *p*<0.05. **Panel B.** A representative Western blot is shown.

Since apolipoproteins play an important role in cholesterol transport, we continued our study by investigating the effect of camphene on apolipoprotein AI, the major and critical protein component of HDL. HepG2 cells were treated with different concentrations of camphene (5, 10, 37 or 50 μM) for 48 h and the protein levels of apoAI were quantified. HepG2 cells retain the ability to synthesize and secrete apoAI as well as other lipoproteins produced by normal hepatocytes and they have been used extensively for studying the factors that regulate hepatic lipoprotein metabolism [41, 42]. It was found that camphene increased apoAI protein expression in HepG2 cells in a concentration-dependent manner. In particular, camphene treatment at low concentrations (1 and 5 μM) had no significant effect on apoAI intracellular protein levels. However, there were significant increases in apoAI protein levels in cells incubated with 10, 37 or 50 μM camphene by 2-fold (*p*<0.05), 3-fold (*p*<0.001) and 4-fold (*p*<0.001) respectively, as compared to control levels (Fig 7) demonstrating that camphene up-regulates apoAI protein expression in a concentration-dependent manner.

**Fig 8:**
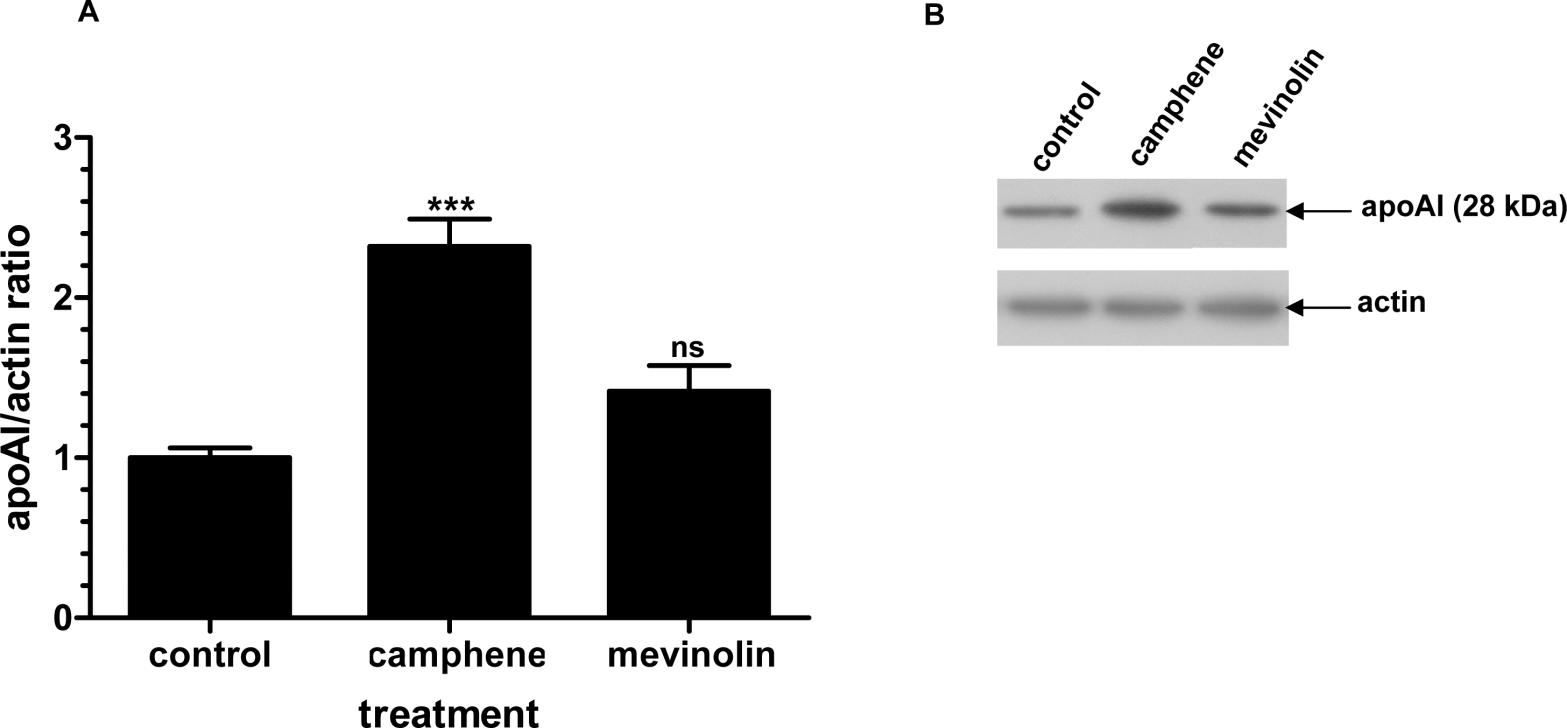
Effect of camphene and mevinolin on the protein expression of apoAI. **Panel A.** On day 7, HepG2 cells were incubated for 48 h with camphene (37 μM) and mevinolin (37 μM) in DMEM containing 10% LPDS. Total cell protein was extracted and 50 μg of protein were separated by SDS PAGE and analyzed by Western immunoblotting using goat anti-apoAI antibody as described. Mouse β-actin was used to control for equal loading and normalization. Relative intensity of the bands was quantified using Phosphorimager. Values express the apoAI/actin ratio and are calculated by comparison to control samples the value of which is defined as 1.Values are means ± SD of three independent experiments in triplicates. *p<*0.001 (***) and ns (non significant) vs control by the Student-Newmann-Keuls test. **Panel B.** A representative Western blot is shown.

To further study the nature of this regulation by camphene, another drug commonly used to repress cholesterol synthesis, mevinolin (37 μM) was used to similar conditions as in the experiments with camphene treatment (37 μM). The protein levels of apoAI in the presence of mevinolin were only slightly altered and mevinolin treatment had no significant effect compared to control cells. Immunoblotting analysis of HepG2 cell lysates revealed that camphene treated cells had a 3-fold (*p*<0.001) increase in apoAI protein levels compared to 1.4-fold increase in cells receiving mevinolin (*p*>0.05) (Fig 8). Therefore, camphene in addition to regulating cholesterol biosynthesis also raises apoAI levels in HepG2 cells.

### Effect of camphene on SREBP-1 and SREBP-2 transcription factors

Since SREBPs are involved in cholesterol and triglyceride synthesis we studied whether the hypolipidemic activity of camphene could be associated to possible changes in SREBP-1 or SREBP-2 protein levels. HepG2 cells were treated for 24 h with camphene (37 μM) and mevinolin (37 μM) in DMEM containing 10% LPDS and SREBP-1 and SREBP-2 protein levels were analyzed by Western blot.

**Fig 9:**
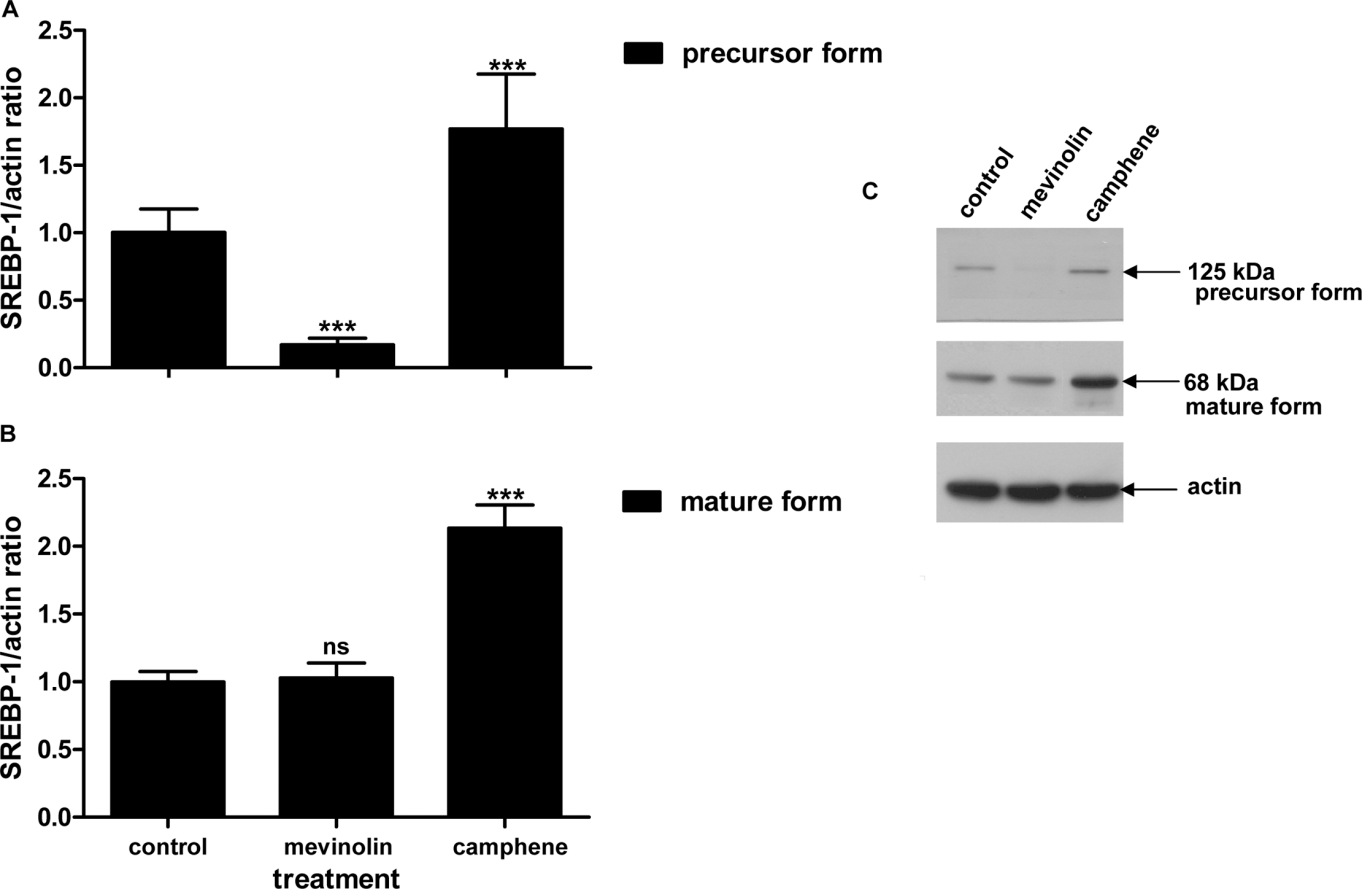
Effect of camphene and mevinolin on the protein expression of SREBP-1. A and B panels. **Effects of the compounds on the mature and the precursor form of SREBP-1 respectively.** On day 7, HepG2 cells were incubated for 24 h with camphene (37 μM) and mevinolin (37 μM) in DMEM containing 10% LPDS. 25 μg/ml ALLN were added for the last 5 h before harvesting, in order to stabilize the short-lived nuclear forms. Total cell protein was extracted and 50 μg of protein were separated on a 8% polyacrylamide gel and analyzed by Western immunoblotting using rabbit anti-SREBP-1 as described. Mouse β-actin was used to control for equal loading and normalization. Relative intensity of the bands was quantified by Phosphorimager. Values express the SREBP-1/actin ratio and are calculated by comparison to control samples the value of which is defined as 1.Values are means ± SD of three independent experiments in triplicates. *p<*0.05 (*); *p<*0.001 (***); *p>*0.05 and ns (non significant) vs control by the Student-Newmann-Keuls test. **Panel C**. A representative Western blot is shown. The upper band is SREBP-1 precursor (125 kDa) and the lower band is mature SREBP-1 (68 kDa).

SREBP-1 was detected by an antibody recognizing both the inactive precursor and the cleaved active mature form of SREBP-1 transcription factor. In addition, this antibody recognizes both SREBP-1a and SREBP-1c isoforms whose transcripts are both present in HepG2 cells; SREBP-1a is predominant to SREBP-1c with a ratio 2:1 [43]. Camphene treatment resulted in a 1.8-fold (*p*<0.001) increase of the precursor form of SREBP-1, whereas mevinolin treatment decreased the inactive precursor form to undetectable levels (decrease by 0.8-fold, *p*<0.001) (Fig 9). Camphene also increased the amount of mature form of SREBP-1 by 2-fold (*p*<0.001) compared to untreated cells. On the other hand, mevinolin did not affect SREBP-1 active form protein levels as it resulted in negligible changes compared to control cells (*p*>0.05) (Fig 9). A representative Western blot of SREBP-1 precursor and mature form is shown in Fig 9. Therefore, camphene treatment increased the conversion of SREBP-1 from its inactive precursor form to its active transcription factor form as it led to a parallel and proportional increase in both forms of SREBP-1 implying the activation of the proteolysis process. Hence, camphene treatment results in the activation of SREBP-1 transcription factor, whereas mevinolin minimizes the protein expression of its precursor form and does not affect its mature form.

**Fig 10:**
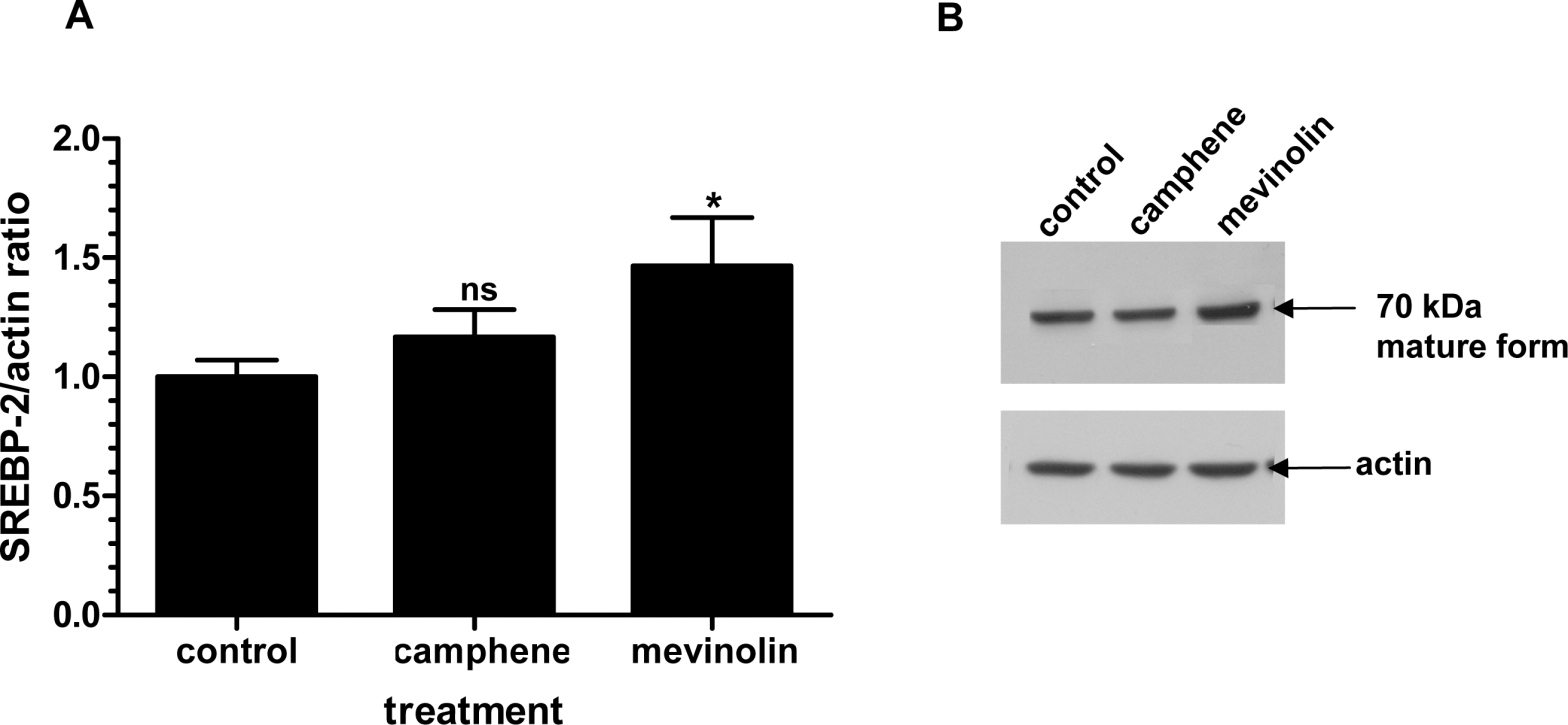
Effect of camphene and mevinolin on the protein expression of the mature form of SREBP-2. **Panel A.** On day 7, HepG2 cells were incubated for 24 h with camphene (37 μM) and mevinolin (37 μM) in DMEM containing 10% LPDS. 25 μg/ml ALLN were added for the last 5 h before harvesting, in order to stabilize the short-lived nuclear forms. Total cell protein was extracted and 50 μg of protein were separated on a 8% polyacrylamide gel and analyzed by Western immunoblotting using rabbit anti-SREBP-2 as described. Mouse β-actin was used to control for equal loading and normalization. Relative intensity of the bands was quantified by Phosphorimager. Values express the SREBP-2/actin ratio and are calculated by comparison to control samples the value of which is defined as 1.Values are means ± SD of three independent experiments in triplicates. *p<*0.05 (*); *p>*0.05 and ns (non significant) vs control by the Student-Newmann-Keuls test. **Panel B.** A representative Western blot is shown. SREBP-2 mature form is about 70 kDa.

SREBP-1 is involved in fatty acid and triglyceride metabolism, whereas SREBP-2 is specific to cholesterol homeostasis and biosynthesis. In order to further investigate the molecular mechanism of hypolipidemic action of camphene we studied the effect of camphene on SREBP-2 protein expression. The effect of camphene was also compared to that of mevinolin which is known to affect the expression of SREBP-2. The antibody used for the detection of SREBP-2 recognized only the mature form of the transcription factor. Camphene (37 μM) treatment did not affect the protein levels of SREBP-2 (*p*>0.05), whereas mevinolin (37 μM) caused an increase by 0.5-fold (*p*<0.05) when compared to untreated HepG2 cells (Fig 10). As detected by Western blotting, camphene increased the amount of mature form of SREBP-1, whereas the expression levels of SREBP-2 were not affected. In contrast, mevinolin targets SREBP-2 expression. These results confirm that camphene exerts its hypolipidemic activity effect through a different molecular mechanism of action than that of statins.

### Effect of camphene on MTP expression

**Fig 11:**
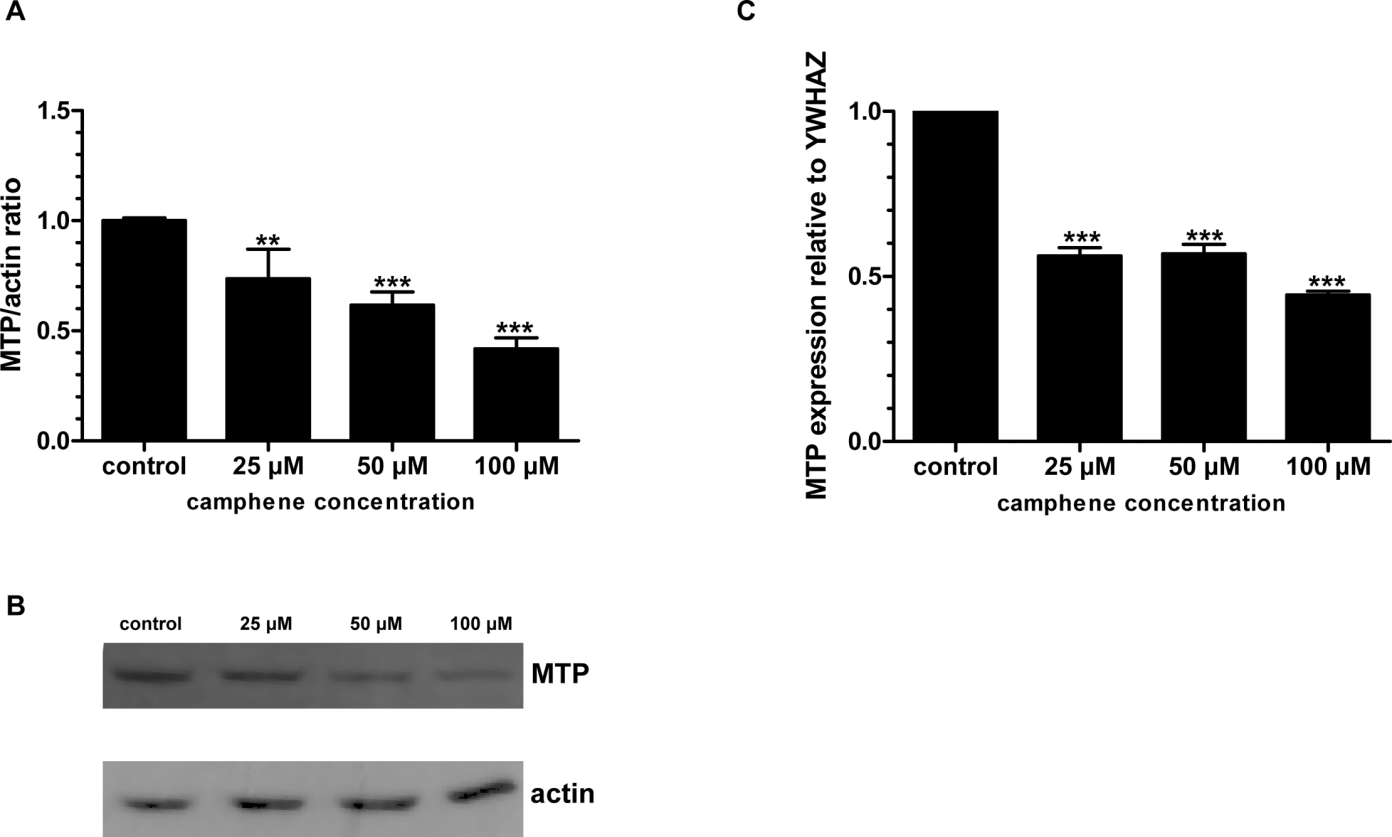
Effect of camphene on MTP expression. On day 7, HepG2 cells were incubated with indicated concentrations of camphene for 24 h in DMEM containing 10% (v/v) human LPDS. **Panel A.** Total cell protein was extracted and 50 μg of protein were separated on a 12% polyacrylamide gel and analyzed by Western immunoblotting using goat anti-MTP as described in Materials and Methods section. Mouse β-actin was used to control for equal loading and normalization. Relative intensity of the bands was quantified by Phosphorimager. Values express the MTP/actin ratio and are calculated by comparison to control samples the value of which is defined as 1. **Panel B.** A representative Western blot is shown. **Panel C.** Total RNA was extracted and subjected to Real Time PCR. The results represent the relative MTP mRNA normalized to endogenous YWHAZ mRNA. The same results were observed after normalization to the housekeeping gene actin (data not shown). The abundance of mRNA in the control-untreated cells was set at 1. Data represent means ± SD of three independent experiments. *p<*0.001 (***) and *p<*0.01 (**) vs control cells by the Student-Newmann-Keuls test.

To test the possibility that camphene might affect MTP expression, MTP mRNA and protein levels in camphene treated HepG2 cells were assessed. Camphene treatment resulted to significant reductions of MTP protein levels by 27 (*p*<0.01), 38 (*p*<0.001) and 52% (*p*<0.001) at concentrations of 25, 50 and 100 μM respectively (Fig 11A and 11B). A significant reduction in the expression levels of MTP mRNA was also observed in HepG2 cells treated with camphene. As shown in Fig 11C, camphene significantly decreased the level of MTP mRNA by 55% (*p*<0.001) compared to control-untreated cells.

## Discussion

Disorders of lipid metabolism are the primary risk factor for cardiovascular disease which remains a leading cause of death and disability. Among the most important risk factors for this disease are not only high total cholesterol levels, but also high triglyceride levels and low levels of HDL cholesterol. The known lipid lowering drugs regulate lipid metabolism by different mechanisms, but have also many side effects [44–46]. Therefore, various pharmacologically active molecules are under investigation in order to find new therapies for dislipidemia [47, 48]. We have recently shown that camphene, a plant derived monoterpene, exhibited a hypolipidemic activity *in vivo* as it decreased total cholesterol, LDL cholesterol and triglycerides in plasma of hyperlipidemic rats, without affecting HMG-CoA reductase activity [12]. In the present study, we provide additional evidence for the hypolipidemic effect *in vitro* and the molecular mechanism of lipid-lowering action of camphene.

In order to evaluate the hypolipidemic activity of camphene we studied its effect on apoAI. Elevated plasma concentrations of apoAI represent a strong negative risk factor for the development of CHD and various experimental manipulations targeting increased production of apoAI are associated with reduced atherogenicity [29, 49, 50]. Thus, upregulation of endogenous apoAI expression is widely considered one of the most promising approaches to the development of new therapies targeted to HDL [51]. In the present study, we demonstrate for the first time that camphene significantly increased apoAI protein expression. Camphene presented a stronger effect to increase apoAI expression as compared with mevinolin treatment, which resulted in a smaller and non significant increase of apoAI levels. Indeed, mevinolin is reported to be less effective than other statins in affecting apoAI synthesis and secretion [52]. Previous studies have shown that treatment with statins in patients with low HDL and elevated triglycerides, has only moderate effect on HDL concentrations, raising HDL cholesterol by an average 5–15% [53]. *In vitro* studies have indicated that this effect may be the result of an increased expression of apoAI [52, 54]. Further studies are required in order to define whether camphene also exerts beneficial effects on HDL cholesterol concentration by stimulating hepatic apoAI expression synthesis or by decreasing either the breakdown or secretion of apoAI.

Since camphene treatment led to the decrease of total cholesterol, LDL cholesterol and triglycerides, we examined its effect on the SREBP transcription factors that are regulators of lipid homeostasis. SREBP-1c primarily regulates genes of fatty acid and triglyceride metabolism, SREBP-2 is mainly responsible for cholesterol related genes and SREBP-1a targets genes involved in both cholesterol and triglyceride pathways [34, 35, 55]. Camphene treatment upregulated the expression of both precursor and mature forms of SREBP-1, implying that camphene triggers the photeolytic process of SREBP-1 precursor form, which then translocates into the nucleus and activates the transcription of target genes. Mevinolin treatment decreased the expression of inactive SREBP-1 precursor form to undetectable levels and had no effect on the expression of the mature form. On the other hand, treatment with mevinolin was paralleled by an increase of SREBP-2 protein (mature form), while camphene had no effect. These results are in agreement with previous studies showing that statins, atorvastatin and mevinolin, increased SREBP-2 expression but not SREBP-1 [56]. It appears that camphene activates the expression of SREBP1 transcription factor while does not substantially alter SREBP-2, indicating that camphene regulates primarily genes involved in fatty acid rather than in cholesterol biosynthesis.

We have previously shown that camphene reduces cholesterol content not only in the plasma of hyperlipidemic rats but also in HepG2 cells [12]. Therefore in the present study we evaluated the effect of camphene on the *de novo* cholesterol biosynthesis in HepG2 cells. Although camphene inhibited the incorporation of [^14^C]-acetate into newly synthesized cholesterol, suggesting the inhibition of cholesterol synthesis in hepatic cells, the maximum inhibition achieved was 39% at a concentration of 100 μM. These results confirmed the hypocholesterolemic effect of camphene observed previously in rats [12]. However, treatment with statins that inhibit HMG-CoA reductase, the rate limiting enzyme in cholesterol biosynthesis, completely suppressed the incorporation of [^14^C]-acetate into newly synthesized cholesterol, as it was expected [56–59]. Reduction of cholesterol by statins, results in a SREBP-2 dependent increase in LDL receptor expression and scavenging of circulating LDL cholesterol [60]. Indeed treatment with statins led to the activation of SREBP-2 (Fig 10) which targets and activates LDL receptor [61], thereby facilitating plasma cholesterol efflux into the cell to maintain cholesterol homeostasis. On the other hand, camphene treatment did not affect SREBP-2 expression (Fig 10) and this result might explain the reduction of cholesterol to a lower extent than statin treatment. However, the decrease of cholesterol levels attenuated by camphene might have activated SREBP-1a which targets genes responsible for cholesterol metabolism such as LDL receptor. Our results indicate that the effect of camphene is most likely regulated through SREBP-1 in response to a decrease in the intracellular cholesterol content.

Previously, camphene significantly reduced plasma triglycerides levels in hyperlipidemic rats [12]. In the present study, camphene also inhibited the incorporation of [^14^C]-acetate into newly synthesized triglycerides and significantly inhibited *de novo* triglycerides biosynthesis by 34% in HepG2 cells (Fig 3). These data indicate that camphene probably lowers plasma triglycerides by reducing the rate of hepatic triglyceride biosynthesis. Interestingly, HMG-CoA inhibitors, atorvastatin and mevinolin, increased the production of newly synthesized triglycerides by 20 and 26% respectively. Previous studies have shown that statins have a heterogeneous effect on the triglyceride levels. They have been shown to increase triglyceride biosynthesis from [^14^C]-acetate [56, 62, 63] or to lower triglycerides in clinical studies [64, 65] and in experimental animals [66]. Therefore, changes in triglyceride levels during statin therapy range between an effective reduction and in same cases of hypertriglyceridemic patients with distinct increases [67]. A possible reason for this is that SREBP genes essential for fatty acid synthesis are linked to the cellular level of cholesterol [68, 69]. Previous studies in transgenic mice overexpressing SREBP-2, have also shown elevated levels of triglycerides [70]. The finding that camphene leads to a potent decrease in triglyceride levels both *in vitro* and *in vivo* suggests its use as a hypotriglyceridemic agent.

Triglyceride synthesis is regulated by cellular processes involved in fatty acid synthesis and their esterification to form triglycerides. We therefore investigated the effect of camphene on the *de novo* fatty acid biosynthesis. Camphene treatment resulted in an inhibitory effect on the *de novo* fatty acid biosynthesis (conversion of acetate to palmitate, Fig 4) by significantly reducing the incorporation of [^14^C]-acetate into fatty acids by 53% (Fig 5). The observation that camphene induces the expression of SREBP-1 which activates genes involved in fatty acid and triglyceride biosynthesis implies that camphene may exert its triglyceride lowering effect through different metabolic processes. We therefore examined whether camphene has a significant role in the esterification of fatty acids to form triglycerides. This study was performed by examining the incorporation of [^14^C]-oleic acid into triglycerides in order to monitor the conversion of acyl-CoA and diacylglycerol to form triglycerides (Fig 4). Acyl CoA:diacylglycerol acyltransferase (DGAT) is the key enzyme of triglyceride synthesis and catalyzes the esterification of fatty acids to form triglycerides. Studies with DGAT deficient mice and DGAT inhibitors showed that reduced activity of this enzyme is translated into reductions in triglycerides [71]. DGAT was not reported as a potential target of SREBP-1 in the microarray analysis of SREBP-1 transgenic mice [72, 73]. Camphene did not alter the esterification of oleic acid to produce triglycerides implying that camphene effect is probably not mediated by DGAT. Thus, camphene decreased triglyceride synthesis from acetate in hepatocytes and inhibited *de novo* fatty acid synthesis but it did not inhibit fatty acid esterification. Thus, our data indicate that camphene reduces triglyceride production by inhibiting fatty acid synthesis from acetate.

A possible mechanism of the hypolipidemic action of camphene may be through the inhibition of MTP. MTP plays a critical role in the assembly and secretion of very low density lipoproteins (VLDL) in the liver and MTP inhibition eliminates the production of atherogenic lipoproteins particles, VLDL and chylomicrons [74]. SREBP-1c has been reported to negatively regulate the expression of MTP [75]. Our results show that camphene treatment reduced MTP expression in both mRNA and protein levels in HepG2 cells. Since camphene upregulated the expression of SREBP-1c, this activation might have led to the observed decreased MTP expression. The MTP promoter contains sterol response elements that promote MTP expression when cellular sterols are increased and suppress MTP expression when their availability is reduced [75, 76]. SREBP-1 is activated under sterol-depleted conditions and therefore upregulation of SREBP-1 as a response to cholesterol lowering of camphene might lead to a negative regulation of MTP. Indeed, MTP inhibitors were shown to reduce triglyceride and cholesterol content *in vivo* and *in vitro* [77–79]. Additionally, camphene treatment resulted in a reduction of fatty acids, the substrate for triglyceride formation, and this could also reflect a decrease in VLDL production. Therefore, camphene reduces triglyceride levels by inhibiting fatty acid synthesis and could mediate its triglyceride lowering effect in hepatocytes through MTP inhibition.

Camphene has a moderate effect on cholesterol biosynthesis in regard with statins, but camphene treatment led to a potent inhibition of triglyceride biosynthesis. Plasma triglyceride levels constitute an important independent risk factor in CHD [80]. Consequently, the synthesis of triglycerides has become a potential target for pharmacological intervention. Pharmaceutical interventions developed for the management of hypertiglyceridemia have been also shown to inhibit triglyceride biosynthesis in HepG2 cells and niacin was reported to reduce the incorporation of [^14^C]-acetate into triglycerides by 20–40% in HepG2 cells [81]. Moreover, camphene increased apolipoprotein AI expression, the major constituent of HDL, indicating that camphene treatment may protect against the adverse effects of dyslipidemia. Today, HDL therapies that lower cardiovascular risk are not yet available [82] and multiple drug classes (fibrates, niacin, statins) induce modest increases in HDL cholesterol concentration. A probable mechanism whereby camphene exerts its hypolipidemic effect is through MTP inhibition. Therefore, camphene could be tested as a natural derived compound for the treatment of dislipidemia. We have previously shown that camphene shows no cytotoxicity in human hepatic cells and the lipid lowering dose decreasing plasma cholesterol and triglycerides in rats was defined at 30mg/kg body weight [12] and it is much lower than the upper limit of camphene in rats which is mentioned to be 5g/kg body weight, (CAS No. 79-92-5: Screening information Data Set (SIDS) of OECD High Volume Chemicals Programme, 1993). It is used as a food additive for artificial flavouring and is listed as a pharmacologically safe compound according to US Food and Drug Administration and individual daily uptake is reported to be 0.05μg/kg/day. The upper limit dosage in humans is recommended to be 20mg/kg body weight by the Council of Europe Committee of Experts on Flavoring Substances [16]. However, pharmacodynamic and pharmacokinetic studies in animals and in humans are required to determine whether camphene might offer advantages over statins and other current therapies of dislipidemia or whether it can be used in a combination therapy for more beneficial effects in the treatment and management of abnormal lipid concentrations. Given the critical role that the management of hyperlipidemia plays in cardiovascular disease, the results of our study provide insights into the development of camphene as a lipid lowering agent.
